# Efficacy of argon-helium cryoablation combined with chemotherapy in the treatment of advanced NSCLC

**DOI:** 10.12669/pjms.40.9.9882

**Published:** 2024-10

**Authors:** Yongfei Guo, Jing Yan, Mao Yang, Tongguo Si

**Affiliations:** 1 Yongfei Guo Department of Invasive Interventional Therapy, Tianjin Cancer Hospital Airport Hospital, Tianjin City, P.R. China; 2 Jing Yan Department of Oral and Maxillofacial Surgery, Armed Forces Specialized Medical Center, Tianjin City, P.R. China; 3 Mao Yang Department of Invasive Interventional Therapy, Tianjin Cancer Hospital Airport Hospital, Tianjin City, P.R. China; 4 Tongguo Si Department of Invasive Interventional Therapy, Tianjin Cancer Hospital Airport Hospital, Tianjin City, P.R. China

**Keywords:** Argon-helium cryoablation, Advanced NSCLC, Chemotherapy

## Abstract

**Objective:**

To explore the efficacy of argon-helium cryoablation (AHC) combined with chemotherapy in the treatment of advanced non-small cell lung cancer (NSCLC).

**Methods:**

A retrospective study was carried out between February 2020 to June 2022 of 85 patients with advanced NSCLC admitted to Tianjin Cancer Hospital Airport Hospital. Patients were categorized into two groups based on whether they had received AHC: patients received chemotherapy treatment alone (chemotherapy group, n=41); patients received chemotherapy combined with AHC (combined group, n=44). Tumor control rate, serum tumor marker levels, quality of life, and median survival time between the two groups were compared.

**Results:**

Tumor control rate in the combined group (86.36%) was significantly higher than that in the chemotherapy group (68.29%) (P<0.05). After treatment, the levels of serum cytokeratin 19 fragment (CYFRA21-1), carcinoembryonic antigen (CEA) and glycoprotein antigen 125 (CA125) in the two groups were significantly lower than those before the treatment, and significantly lower in the combined group compared to the chemotherapy group (P<0.05). After the treatment, the quality of life of patients in both groups was significantly higher than before the treatment. Quality of life in the combined group was significantly higher than in the chemotherapy group (P<0.05). One year after treatment, the median survival time of the combined group (10.5 months; 95% CI: 9.775-11.225) was significantly higher than that of the chemotherapy group (9.4 months; 95% CI: 8.55-10.323) (P=0.045).

**Conclusions:**

Compared with chemotherapy alone, conventional chemotherapy combined with AHC in the treatment of advanced NSCLC can significantly reduce the levels of serum tumor markers and improve overall treatment efficacy, quality of life and 1-year overall survival rate.

## INTRODUCTION

Lung cancer (LC) is the leading cause of cancer-related deaths worldwide, of which approximately 80%-85% are non-small cell lung cancer (NSCLC).^1^,^2^ The main subtypes of NSCLC are adenocarcinoma (40%), squamous cell carcinoma (25%), and large cell carcinoma (10%).^3^ Due to the lack of symptoms or non-specific manifestations in the early stage of LC, the early diagnosis of LC is low. About 70% of patients are diagnosed at an advanced stage and cannot be undergo radical surgery. Therefore, these patients require conservative management such as chemotherapy and radiotherapy.^4^,^5^

Chemotherapy is commonly used in advanced NSCLC, but the overall effect of individual treatment is not good, and the incidence of toxic side effects is high.^6^,^7^ Argon-helium cryoablation (AHC) is a new minimally invasive and safe local treatment method for malignant tumors, which is based on argon and helium as refrigerants and heating agents, and uses rapid heating and ultra-low temperature freezing to damage local tumor tissue.^8^,^9^

Although studies have reported that AHC combined with chemotherapy can produce synergistic effects, most focus on complications, survival rate, or remission rate. In this study, we retrospectively analyzed the treatment status of patients with advanced NSCLC received AHC combined with chemotherapy from the perspective of remission rate, serum tumor marker levels, quality of life, and survival to assess the efficacy of the combined regimen in the treatment of advanced NSCLC.

## METHODS

A retrospective study was carried out between February 2020 to June 2022 of 85 patients with advanced-stage lung cancer admitted to Tianjin Cancer Hospital Airport Hospital. Patients were categorized into two groups based on whether they had received AHC: patients received chemotherapy treatment alone (chemotherapy group, n=41); patients received chemotherapy combined with AHC (combined group, n=44).

### Ethical Approval:

The Medical Ethics Committee of Tianjin Cancer Hospital Airport Hospital approved this study with number LWK-2023-0003, Date: November 9^th^ 2023.

### Inclusion criteria:

- Patients with histologically or pathologically confirmed NSCLC.^10^
- Patients with stage IIIB - IV NSCLC who could not undergo standard surgical treatment.
- Age<80 years old.
- Complete clinical data.
- Karnofsky Performance Scale score ≥ 60 points, with an estimated survival time >3 months.

### Exclusion criteria:

- Patients with pulmonary tuberculosis, pulmonary infections, and other lung diseases.
- Patients with other benign and malignant tumors.
- Patients with hematopoietic dysfunction or immune deficiency.
- Patients who received radiotherapy and chemotherapy treatment within six months prior to inclusion in the study.
- If the distance between the edge of the tumor and the bronchus is less than 5 cm.

Patients in the chemotherapy group received conventional chemotherapy regimen, while patients in the combined group received AHC first and then chemotherapy 3-5 days after AHC. Below are the treatment approach of chemotherapy and AHC.

***1) Conventional chemotherapy regimen:*** On the first day, intravenous infusion of docetaxel (Henan Province Guoyao Pharmaceutical Group Co., Ltd., specification: 0.5 ml: 20mg) 75 mg/m^2^; Intravenous drip of cisplatin (Qilu Pharmaceutical Co., Ltd., specification: 10 mg) 30 mg/m^2^ on the 1st to 3rd day; Oral administration of 7.5mg dexamethasone (Xi’an Lijun Pharmaceutical Co., Ltd., specification: 0.75mg) one day before and on the day of intravenous infusion of docetaxel, and one day after chemotherapy, twice a day. One cycle lasts for 21 days, with a total of four cycles per treatment.

***2) AHC procedures:*** Preoperative enhanced CT (Siemens, Germany) scanning examination was done to clarify the location, volume, morphology, and distribution of peripheral blood vessels and nerves of the lesion. Using an 8-knife independent cryotherapy device (CRYOCARE, USA), the surgical puncture point was marked and processed according to the CT scanning results. Local anesthesia on the puncture site and surrounding tissues was performed, and the puncture needle was sent to the reserved area at the puncture point. An expansion tube and guide wire were inserted, the puncture channel was dilated, a catheter sheath and an argon helium knife were inserted. The catheter sheath was then removed, and the ultra-low temperature freezing system was initiated to implement rapid freezing (temperature reduced to -130 °C within 30 seconds). Freezing continue for 20 minutes, after which it was terminated and the heating system started. After the temperature rose to 0°C, heating was continued to 40°C (for 20 minutes). A freeze-thaw cycle was then repeated for the second time. The operator ensured that that the ice hockey ball completely covered the tumor lesion, and the argon helium knife was removed.

### Observation indicators:

- ***Tumor control status:*** Evaluation was based on RECIST criteria.^11^ Complete remission was achieved when the lesion was known to have disappeared and persisted for ≥ 4 weeks; Partial remission occurred when the sum of the maximum single paths of the tumor lesion decreased by more than 30% and lasted for ≥ 4 weeks; Progression occurred if the sum of the maximum single paths of the tumor lesion increased by more than 20% or there was a new lesion; The rest of the conditions were deemed stable. Complete remission, partial remission, and stability were included in the tumor control rate.^12^
- ***Serum tumor markers:*** Briefly, levels of keratin 19 soluble fragment (CYFRA21-1), carcinoembryonic antigen (CEA) and carcinoembryonic antigen 125 (CA125) in the serum of fasting peripheral blood were detected by enzyme-linked immunosorbent assay using Hitachi 7600-020 fully automatic biochemical analyzer, and the reagent kit from Wuhan Doctoral Biotechnology Co., Ltd.
- ***Quality of life:*** Evaluate according to the Quality of Life Scale for Cancer Patients (EORTC QLQ-C30), including functional areas (15 items, 15-60 points), overall health status areas (2 items, 2-14 points), and symptom areas (13 items, 13-52 points). Higher overall health status and functional score indicated better quality of life, while the higher symptom score correlates with the worse quality of life.^13^
- ***Prognostic situation:*** survival after one year of the treatment.

### Statistical Analysis:

All data analysis was conducted using SPSS26.0 software (IBM Corp, Armonk, NY, USA). Normality of the data was assessed using the Shapiro-Wilk test and expressed as mean ± standard deviation. Independent sample t test was used for inter group comparison, and paired *t* test was used for intra group comparison. Data of non-normal distribution were expressed as median and interquartile interval, Mann-Whitney *U* test was used for inter group comparison, and Wilcoxon signed rank test was used for intra group comparison. The counting data were represented by n(%) and compared using Chi-squared test. Survival analysis was conducted using the Kaplan-Meieri method and compared statistically using the Log rank test. P<0.05 indicated statistically significant difference.

## RESULTS

A total of 85 patients met the inclusion criteria of the study. Age of the patients ranged from 49 to 79 years, with a mean age of 64.01 ± 7.66 years. A total of 52 patients were in stage III-b, 33 patients were in stage IV. Body mass index (BMI) ranged from 17.9 to 28.6kg/m^2^, with a mean of 23.47 ± 2.63kg/m^2^. There was no significant difference in the baseline data between the two groups (P>0.05), Table-I. The tumor control rate of the combined group (86.36%) was higher than that of the chemotherapy group (68.29%) (P<0.05), Table-II.

**Table-I:** Comparison of two groups of baseline data.

Group	Gender (Male/female)	Age (years)	Pathologic staging (IIIb/IV)	Pathological type (Adenocarcinoma/Squamous cell carcinoma)	BMI (kg/m^2^)	Education level (below high school/above high school)
Combined group (n=44)	30/14	63.75±7.49	28/16	27/17	23.19±2.56	27/17
Chemotherapy group (n=41)	21/20	64.29±7.93	24/17	21/20	23.77±2.72	29/12
*χ*^2^/*t*	2.544	-0.325	0.232	0.888	-1.013	0.829
*P*	0.11	0.746	0.630	0.346	0.314	0.363

BMI: body mass index.

**Table-II:** Comparison of tumor control rates between the groups.

Group	Complete remission	Partial remission	Stability	Progression	Total effective rate
Combined group (n=44)	3 (6.82)	19 (43.18)	16 (36.36)	6 (13.64)	38 (86.36)
Chemotherapy group (n=41)	1 (2.44)	14 (34.15)	13 (31.71)	13 (31.71)	28 (68.29)
*χ* ^2^					3.993
*P*					0.046

There was no significant difference in the levels of CYFRA21-1, CA125, and CEA between the two groups before the treatment (P>0.05). After the treatment, levels of CYFRA21-1, CA125, and CEA in both groups decreased compared to pretreatment levels, and were significantly lower in the combined group (P<0.05), Table-III.

**Table-III:** Comparison of serum tumor marker levels between the groups.

Time	Group	CYFRA21-1 (ng/ml)	CA125 (U/ml)	CEA (ng/ml)
Before treatment	Combined group (n=44)	6.15±1.01	42.70±7.09	8.48±0.83
	Chemotherapy group (n=41)	6.31±0.92	43.68±6.52	8.20±1.12
	*t*	-0.738	-0.661	1.337
	*P*	0.462	0.510	0.185
After treatment	Combined group (n=44)	3.68±1.00^a^	23.55±6.73^a^	4.45±0.86^a^
	Chemotherapy group (n=41)	4.54±0.91^a^	31.54±6.43^a^	6.12±1.10^a^
	*t*	-4.134	-5.589	-7.845
	*P*	<0.001	<0.001	<0.001

***Note:*** Compared with the same group before treatment,

a P<0.05 CYFRA21-1: cytokeratin 19 soluble fragment; CEA: carcinoembryonic antigen; CA125: cancer antigen 125.

Before the treatment, there was no significant difference in the scores of overall health status, functional areas, and symptom areas between the two groups (P>0.05). After the treatment, the overall health status, and functional scores of the two groups increased compared to pretreatment levels, and were significantly higher in the combined group. The score in the symptom domain decreased compared to before the treatment, and was significantly lower in the combined group (P<0.05), Table-IV.

**Table-IV:** Comparison of quality of life between the groups.

Time	Group	Overall health status	Functional scores	Symptom domain
Before treatment	Combined group (n=44)	7 (5.5, 8)	34.55±4.99	32.43±5.82
	Chemotherapy group (n=41)	7(6, 8)	35.54±6.28	34.24±5.99
	*t*/*Z*	-0.600	-0.809	-1.414
	*P*	0.549	0.421	0.161
After treatment	Combined group (n=44)	10(9, 11.5)^a^	46.32±5.73^a^	22.25±5.12^a^
	Chemotherapy group (n=41)	9(8, 9)^a^	42.63±5.97^a^	29.10±5.60^a^
	*t/Z*	-3.866	2.903	-5.891
	*P*	<0.001	0.005	<0.001

***Note:*** Compared with the same group before treatment,

a P<0.05.

**Fig.1:**
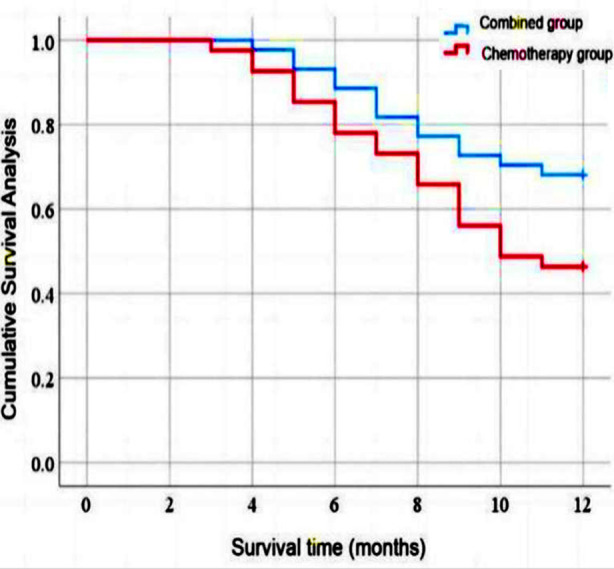
Comparison of survival curves between the groups.

One year after treatment, the median survival time of the combined group (10.5 months; 95% CI: 9.775-11.225) was significantly higher than that of the chemotherapy group (9.4 months; 95% CI: 8.55-10.323) (*P*=0.045), Fig.1.

## DISCUSSION

This study indicated that the combination of conventional chemotherapy and AHC in the treatment of advanced NSCLC has high practical value, and is efficient in downregulating the levels of tumor markers, improving treatment effectiveness, improving patient quality of life, and prolonging survival cycle.

Argon-helium knife was developed and first applied in the late 20th century and has been widely used in the treatment of mid to late- stage LC as a treatment method that has a good effect and is inexpensive, safe, and suitable for patients who would not tolerate surgery.^8^,^9^,^14^ A study by Jiang W et al.^14^ has shown that the combination of chemotherapy and argon helium knife therapy for NSCLC is feasible and effective, and is beneficial for improving the therapeutic effect of the disease. Moreover, combining argon helium knife and chemotherapy in patients with advanced NSCLC was able to prolong the survival cycle, and improve quality of life. These results are consistent with the conclusions of our study.

Cheng R et al.^15^ showed that compared with radiofrequency ablation, AHC did not show significant differences in immune function of LC patients, but was associated with a lower incidence of complications. Some studies have shown that radiotherapy can prolong the life cycle of LC patients to a certain extent, but is accompanied by serious side effects, including bone marrow suppression, liver function damage, immune function suppression, leukopenia and gastrointestinal reactions, and is affected by low autoimmune function and weak constitution, resulting in poor treatment effect.^16^-^18^ AHC, on the other hand, can utilize low temperature to crystallize and expand water in tumor cells, and gradually cause cell rupture upon rewarming. The alternating effect of high and low temperature can damage the blood supply and cause ischemic necrosis of tumor cell supply vessels.^9^,^14^ Necrotic tumor tissue, in turn, may trigger the production of anti-tumor cold immune antibodies, thereby strengthening the body’s anti-tumor ability and inhibiting the growth of tumor lesions.^14^,^19^ Zhou Q et al.^20^ conducted a retrospective analysis of the clinical data of late-stage LC patients receiving AHC treatment, and found that their disease control rates were satisfactory at three months, six months, and twelve months after the treatment, with 93.6%, 87.0%, and 75.6%, respectively. The one-, two-, and three- year survival rates were 85.1%, 42.6%, and 27.6%, respectively, confirming that AHC treatment for late-stage LC is safe and effective.

The meta-analysis by Duan H et al.^21^ on the effectiveness and safety of AHC in the treatment of advanced NSCLC also confirmed that AHC combined with chemotherapy has satisfactory safety, and can improve short-term and long-term treatment effects, as well as immune function, and quality of life of LC patients. Our results further confirm these reports. For the late-stage LC, chemotherapy is generally the main treatment mode. However, chemotherapy alone has relatively low benefit rate and is associated with significant adverse reactions and reduced quality of life.^22^,^23^ The results of our study show that AHC combined with chemotherapy can significantly improve the quality of life and prolong survival time after the treatment.

### Limitations:

This is a single center retrospective study with a small sample size and selection bias. The follow-up time was short, and there was no investigation of the immune function status of late-stage LC patients before and after the treatment. Further research is needed to confirm the results of this study.

## CONCLUSION

Compared with chemotherapy alone, the combination of conventional chemotherapy and AHC in the treatment of advanced NSCLC can reduce the levels of serum tumor markers and improve overall treatment efficacy, quality of life and 1-year overall survival rate.

### Authors’ contributions:

**YG** and **JY:** Conceived and designed the study.

**YG**, **JY**, **MY** and **TS:** Collected the data and performed the analysis.

**YG** and **JY:** Were involved in the writing of the manuscript and is responsible for the integrity of the study.

All authors have read and approved the final manuscript.
